# Expression of APOBEC3 Lentiviral Restriction Factors in Cats

**DOI:** 10.3390/v11090831

**Published:** 2019-09-07

**Authors:** Ryan M. Troyer, Jennifer L. Malmberg, Xin Zheng, Craig Miller, Martha MacMillan, Wendy S. Sprague, Britta A. Wood, Sue VandeWoude

**Affiliations:** 1 Department of Microbiology, Immunology and Pathology, Colorado State University, Fort Collins, CO 80523, USA (J.L.M.) (X.Z.) (C.M.) (M.M.) (W.S.S.) (B.A.W.) (S.V.); 2 Department of Microbiology and Immunology, University of Western Ontario, 1151 Richmond St., London, ON N6A 5C1, Canada; 3 Wyoming State Veterinary Laboratory, University of Wyoming, 1174 Snowy Range Rd., Laramie, WY 82072, USA; 4 Department of Veterinary Pathobiology, Oklahoma State University, Stillwater, OK 74078, USA; 5 Sprague Medical and Scientific Communications, LLC, Fort Collins, CO 80528, USA; 6 The Pirbright Institute, Pirbright, Surrey GU24 0NF, UK

**Keywords:** APOBEC, feline, feline immunodeficiency virus, FIV, lentivirus, mRNA

## Abstract

Feline immunodeficiency virus (FIV) is a naturally occurring T-cell tropic lentiviral disease of felids with many similarities to HIV/AIDS in humans. Similar to primate lentiviral-host interactions, feline APOBEC3 (A3) has been shown to inhibit FIV infection in a host-specific manner and feline A3 degradation is mediated by FIV Vif. Further, infection of felids with non-native FIV strains results in restricted viral replication in both experimental and naturally occurring infections. However, the link between molecular A3-Vif interactions and A3 biological activity during FIV infection has not been well characterized. We thus examined expression of the feline A3 genes A3Z2, A3Z3 and A3Z2-Z3 during experimental infection of domestic cats with host-adapted domestic cat FIV (referred to as FIV) and non-adapted *Puma concolor* FIV (referred to as puma lentivirus, PLV). We determined A3 expression in different tissues and blood cells from uninfected, FIV-infected, PLV-infected and FIV/PLV co-infected cats; and in purified blood cell subpopulations from FIV-infected and uninfected cats. Additionally, we evaluated regulation of A3 expression by cytokines, mitogens, and FIV infection in cultured cells. In all feline cells and tissues studied, there was a striking difference in expression between the A3 genes which encode FIV inhibitors, with A3Z3 mRNA abundance exceeding that of A3Z2-Z3 by 300-fold or more. Interferon-alpha treatment of cat T cells resulted in upregulation of A3 expression, while treatment with interferon-gamma enhanced expression in cat cell lines. In cats, secondary lymphoid organs and peripheral blood mononuclear cells (PBMC) had the highest basal A3 expression levels and A3 genes were differentially expressed among blood T cells, B cells, and monocytes. Acute FIV and PLV infection of cats, and FIV infection of primary PBMC resulted in no detectable change in A3 expression with the exception of significantly elevated A3 expression in the thymus, the site of highest FIV replication. We conclude that cat A3 expression is regulated by cytokine treatment but, by and large, lentiviral infection did not appear to alter expression. Differences in A3 expression in different blood cell subsets did not appear to impact FIV viral replication kinetics within these cells. Furthermore, the relative abundance of A3Z3 mRNA compared to A3Z2-Z3 suggests that A3Z3 may be the major active anti-lentiviral APOBEC3 gene product in domestic cats.

## 1. Introduction

Apolipoprotein B mRNA-editing enzyme, catalytic polypeptide-like type 3 (APOBEC3 or A3) proteins comprise a family of mammalian cytosine deaminases best known for their robust ability to restrict lentiviral infection. Broadly speaking, A3 proteins bind to single-stranded DNA or RNA and enzymatically edit nucleic acids of the target substrate. Antiretroviral activity of A3 is conferred by the incorporation of cytosine to uracil (C-to-U) mutations in the template strand of cDNA during reverse transcription of the viral genome, ultimately resulting in guanine-to-adenine (G-to-A) mutations in nascent proviral DNA [1,2,3,4,5,6]. Additional non-editing mechanisms of retroviral restriction, including impedance of viral reverse transcriptase activity, have also been reported [7,8,9,10]. To counteract the intrinsic defense mechanism(s) of A3, most lentiviruses have evolutionarily adapted the expression of viral infectivity factor (Vif), which prevents incorporation of host-derived A3 proteins into progeny virions during encapsidation [11,12,13,14]. The molecular interactions between host A3 and lentiviral Vif are well-studied [15,16,17]; however, questions remain regarding substrate specificity and regulation of A3 expression during the intrinsic immune response.

Expression of A3 genes is constitutive in many cell types and tissues, and is not limited to those of innate immune function [18]. Differential expression of A3 subsets, however, can be induced through multiple activation pathways that stimulate distinct A3 genes in cell type-specific patterns [19,20,21,22]. The molecular mechanisms underlying modulation of A3 expression are complex, and have been most thoroughly investigated in the context of human cancers [23] and the innate immune response to HIV [24,25]. For instance, the modulatory capacity of cytokines comprising the interferon (IFN) family is well described and corresponds to enhanced expression of A3 following viral infection [26]. Specifically, enhanced A3 expression and activity has been induced by treatment with IFN-α, corresponding to a measurable decline in HIV replication, as well as increased frequency of G-to-A mutations [27,28]. Additional mechanisms have been elucidated for indirect IFN induction [29], as well as IFN-independent activation. For example, endogenous CCL2 negatively regulates A3 expression in monocyte-derived macrophages in an IFN-independent manner [30], while a direct increase in T cell A3 expression has been documented in response to mitogenic stimulation with phorbol myristate acetate (PMA) and phytohemagglutinin (PHA) [21].

Pressures imposed by viruses and retroelements have shaped the genomic structure of the mammalian A3 locus in a species-specific manner illustrated by various gene duplications, losses, and polymorphisms [31]. Such changes correspond to a gain of novel function(s) that comprise the mammalian antiviral restriction system, exemplifying convergent evolution driven by the arms race between host and virus [32]. All proteins of the A3 family are characterized by distinct zinc-binding domains and can be classified accordingly based on catalytic motif (A3Z1, A3Z2, or A3Z3) [33,34]. The genome of the *Felidae* family is characterized by three copies of A3Z2 (A3Z2a, A3Z2b and A3Z3c), a single copy of A3Z3, and a notable absence of the A3Z1 gene observed in the canine counterpart of the order *Carnivora* [31,35]. An additional transcript containing a ‘linker domain’ is produced via read-through transcription and alternate splicing, resulting in the double domain protein A3Z2-Z3. Variants A3Z2b-Z3 and A3Z2c-Z3 have been identified [35].

Domestic cats (*Felis catus*) and other members of the *Felidae* family are susceptible to a number of retroviruses, including feline immunodeficiency virus (FIV) of the *Lentivirus* genus, feline leukemia virus (FeLV) of the *Gammaretrovirus* genus, and feline foamy virus (FFV) of the *Felispumavirus* genus. Adaptations to evade A3 activity have been elucidated for FIV and FFV. Accessory proteins Vif and Bet oppose A3 restriction to permit FIV and FFV infection, respectively [35,36,37,38,39]. Similar to HIV Vif, FIV Vif targets A3 for poly-ubiquitination and degradation through recruitment to an E3 ubiquitin ligase complex [40]. In contrast, FFV Bet evades A3 restriction via a degradation-independent pathway involving putative formation of insoluble Bet-A3 complexes to circumvent virion encapsidation of A3 [36,37,38]. While anti-FIV activity is conferred by A3Z3 and A3Z2-Z3 [35,39,41,42], anti-FFV activity is primarily attributed to A3Z2 (a-c) [36,37]. A3Z3 and A3Z2-Z3 have a lesser impact on the infectivity of Bet-deficient FFV [36,37]. Interestingly, a counter mechanism directed against A3 activity has not been identified for FeLV, despite the finding that A3Z2-Z3 significantly reduces FeLV infectivity in vitro [35]. A mild inhibitory effect on FeLV infectivity has been demonstrated for A3Z3, while A3Z2(a-c) does not alter infectivity [35]. It has been hypothesized that FeLV may evade A3 activity via a tropism for cells with low A3Z2-Z3 activity, as has been proposed for equine infectious anemia virus (EIAV), the only lentivirus lacking the Vif protein [39].

The activity of retroviral proteins against cellular A3 is typified by species-specific interactions resulting from virus adaptation to a distinct primary host [43,44,45,46]. As such, Vif specificity for A3 represents a barrier to potential cross-species virus transmission [43,46]. Exceptions to this convention, however, are surprisingly common and of notable significance in lentiviral evolution [47]. For instance, interactions between Vif of simian immunodeficiency virus (SIV) and human A3 haplotypes significantly influenced the outcome of spillover infections that marked the inception of the HIV-1 pandemic [16,43]. Despite such implications, the evolutionary pathways of lentiviral adaptation to A3 repertoires of target and non-target hosts are only partially understood [48]. Several studies have documented restriction of HIV-1 by feline A3s, with A3Z2-Z3 conferring the greatest antiviral activity [35,47,49,50]. Interestingly, escape of feline A3 hypermutation by virtue of Vif competence has been documented for SIV derived from macaques (SIVmac) [41]. Within the *Felidae*, FIV spillover infections from bobcats (*Lynx rufus*) to pumas (*Puma concolor*) have been reported, indicating a relaxation of A3-imposed host barriers between closely related feline species [51,52]. Further, several studies have shown that domestic cat FIV Vif is successful in opposing A3 activity of most species of larger cats, demonstrating a generalist adaptation of domestic cat FIV [39]. In contrast, domestic cat infection with FIV of pumas (puma lentivirus, PLV), is abortive and displays signatures of A3 restriction including classic G-to-A hypermutation [53].

PLV infection in domestic cats has been shown to modulate subsequent FIV infection and disease progression [54,55]. First, infection of domestic cats with PLV prior to FIV exposure safeguards against the marked CD4+ T-cell depletion that is characteristic of FIV infection [54]. Second, prior infection with PLV has been shown to dramatically reduce intrahost diversity of FIV through a genetic bottleneck [55]. Finally, alterations in innate immune system parameters have been documented in response to PLV-FIV co-infection, including elevations in cytokines such as IFN-γ and increased numbers of circulating cells of innate immunophenotypes [56,57]. In contrast, comparable measures in the adaptive arm of the immune system have not been identified. Collectively, these findings suggest that innate immunity largely determines the outcome of lentiviral co-infection. We, therefore, hypothesized that increased A3 expression mediates PLV-induced resistance to FIV. To test this hypothesis, we determined A3 expression levels during experimental infection of domestic cats with host-adapted FIV and non-adapted PLV, and compared A3 expression across cell and tissue types. We further investigated regulation of A3 expression by FIV infection, cytokines, and mitogen stimulation in vitro.

## 2. Materials and Methods

### 2.1. Animals

Cats were housed in an AAALAC International accredited, specific-pathogen-free animal facility at Colorado State University (CSU). All procedures were approved by the CSU Animal Care and Use Committee prior to initiation (approval number: 01-246A-08, 1 March 2008).

### 2.2. Cells and Culture Conditions

Primary feline peripheral blood mononuclear cells (PBMC) were purified from blood samples obtained from specific-pathogen-free adult cats in a breeding colony at Colorado State University. PBMCs were isolated on a Histopaque-1077 (Sigma-Aldrich, St. Louis, MO, USA) gradient and washed with phosphate-buffered saline (PBS). In addition to primary PBMCs, three immortalized feline cell lines were used: Crandell-Rees feline kidney cells (CRFK) [58], Mya-1 feline T-lymphoblastoid cells [59], and 3201 feline T-cell lymphoma cells [60]. CRFK cells were grown at 37 °C in 5% CO_2_ in Dulbecco’s modified Eagle’s medium (DMEM) with GlutaMAX-1, 1 g/L d-glucose, and 110 mg/L sodium pyruvate (Thermo Fisher Scientific, Waltham, MA, USA) supplemented with 10% fetal bovine serum (FBS; GE Healthcare Life Sciences, Marlborough, MA, USA), 0.075% sodium bicarbonate, 0.1 mM minimal essential medium (MEM) nonessential amino acids, and 1X penicillin-streptomycin (10,000 U/L penicillin and 10,000 µg/L streptomycin; Thermo Fisher). Primary feline PBMCs, Mya-1 cells, and 3201 cells were grown at 37 °C in 5% CO_2_ in RPMI 1640 medium with GlutaMAX-1 (Thermo Fisher) supplemented with the following: 20% FBS, 9 g/L D-glucose (Sigma-Aldrich), 1X penicillin-streptomycin, 0.075% sodium bicarbonate, 0.1 mM MEM nonessential amino acids, 1 mM sodium pyruvate, and 0.055 mM 2-mercaptoethanol (Thermo Fisher). For PBMCs and Mya-1 cells, 10 ng/mL recombinant human interleukin-2 (MilliporeSigma, Burlington, MA, USA) was also added to the media.

### 2.3. Viruses

All experiments involving feline immunodeficiency virus (FIV) were conducted using the domestic cat FIV-C36 strain [61]. FIV-C36 viral stocks were generated by propagation in Mya-1 cells. All experiments involving puma lentivirus (PLV) used the PLV-1695 strain of FIV-Pco (*Puma concolor*) subtype B, originally derived by culturing PBMCs from a naturally infected British Columbia puma [62]. The PLV-1695 stock used to infect cats in this study was further propagated in Mya-1 cells [63].

### 2.4. RNA and DNA Extractions and cDNA Synthesis

RNA was extracted from cultured cells and PBMCs using TRIzol reagent (Thermo Fisher) according to the manufacturer’s instructions. Any remaining DNA was eliminated by treatment with 2 U of amplification grade DNase I (Thermo Fisher) in a 50 µL volume. RNA preparations were further purified using the RNA cleanup protocol of the RNeasy minikit (Qiagen, Valencia, CA, USA) and quantified using an ND-1000 spectrophotometer (NanoDrop Technologies, Wilmington, DE, USA). RNA was extracted from tissues using the FastRNA Pro Green kit with FastPrep-24 homogenizer following the manufacturer’s protocol (M.P. Biomedicals, Santa Ana, CA, USA). Briefly, 100 mg tissue was homogenized in RNApro solution and Lysing Matrix D using the FastPrep-24 instrument for 40 s at a setting of 6.0. RNA was then purified according to the manufacturer’s instructions, quantified, and stored at −80 °C. Cellular RNA was converted to cDNA according to the manufacturer’s instructions for Superscript II (Thermo Fisher) using random hexamer primers.

### 2.5. Quantification of Feline APOBEC3 Gene Expression by qPCR

Feline APOBEC3 (A3) cDNA was quantified by qPCR using primers specific for A3Z2, A3Z3, and A3Z2-Z3. Primer sequences and reaction details have been published previously [64]. Briefly, the A3Z2 primers detect the expression of all three feline A3Z2 isoforms (A3Z2a, A3Z2b, and A3Z2c) collectively. The A3Z2-Z3 primers detect the expression of all A3Z2-Z3 variants (A3Z2b-Z3, A3Z2c-Z3, and splice variants). An A3Z2b-Z3 plasmid standard curve was used to determine copy number for all three A3 qPCRs. Since the A3Z2 and A3Z3 primers also detect A3Z2-Z3 expression, we subtracted the A3Z2-Z3 copy number from the A3Z2 and A3Z3 copy numbers for each sample. The validity of this subtractive method is supported by the use of a single A3Z2b-Z3 plasmid standard curve for all A3 qPCRs and qPCR efficiencies that were consistently in the 95%-to-100% range for these assays. A3 mRNA copy numbers were normalized to glyceraldehyde-3-phosphate dehydrogenase (GAPDH) expression using a previously described qPCR assay for feline GAPDH [65] with a feline-GAPDH-encoding plasmid standard curve. All primer pairs span exon junctions, and melt curves run on all qPCRs showed single-amplification products, demonstrating target specificity for all assays.

### 2.6. Assessment of Cytokine and Mitogen Effects on APOBEC3 Gene Expression

For cytokine and mitogen stimulation experiments involving suspension cell cultures, 2 × 10^6^ Mya-1 cells were suspended in 2 mL media in 12-well plates and 2 × 10^6^ PBMCs were suspended in 1 mL media in 24-well plates. For adherent CRFK cells, 1 × 10^6^ cells were plated in 5 mL media in 6 cm dishes and allowed to adhere overnight. Replicate wells of Mya-1 (*n* = 3), CRFK (*n* = 3), and PBMC (*n* = 5) were treated with 10 or 100 ng/mL recombinant feline interferon-gamma (IFNγ; R&D Systems, Minneapolis, MN, USA), 100 or 1000 U/mL feline IFN-alpha (IFNα; PBL Biomedical Laboratories, Piscataway, NJ, USA), 5 µg/mL concanavalin A (ConA; Sigma-Aldrich), or 5 µg/mL phytohemagglutinin (PHA; Sigma-Aldrich). At 24 h post treatment, RNA was extracted from cells, as described in Section 2.4.

### 2.7. In Vitro FIV Infection

PMBCs were isolated from three specific pathogen-free cats and cultured at 2 × 10^6^ cells per mL in media containing 5 µg/mL ConA. After 3 days of ConA treatment, PBMC were readjusted to 2 × 10^6^ cells/mL in fresh media without ConA and infected with FIV-C36 at an MOI of 0.001 or mock-infected with media. At days 4, 7, 10, and 14 post-infection, RNA was extracted from triplicate wells (one from PBMC of each cat) for FIV-infected and mock-infected cells, as described in Section 2.4.

### 2.8. Assessment of APOBEC3 Gene Expression and FIV Infection in Cat Blood Cell Subtypes

Three specific pathogen-free cats from a breeding colony at Colorado State University were given an intravenous injection with 10^5^ 50% tissue culture infectious doses (TCID_50_) of FIV-C36 and three cats were sham-infected with uninfected Mya-1 cell supernatant. At 18 weeks post infection (chronic infection phase), approximately 45 mL blood was drawn from all cats and PBMCs were isolated via Histopaque-1077 (Sigma-Aldrich) gradient. PBMCs from each cat were suspended in phosphate buffered saline (PBS) with 3% FBS and divided into two portions for labelling with two panels of antibodies: (1) anti-feline CD4-PE (clone 3-4F4, Southern Biotech, Birmingham, AL, USA) and anti-mouse (feline cross-reactive) B220-FITC (clone RA3-6B2, BD Biosciences, San Jose, CA, USA), and (2) anti-feline CD8-FITC (clone fCD8, Southern Biotech) and anti-human (feline cross-reactive) CD14-Alexa647 (clone TÜK4, Bio-Rad, Hercules, CA, USA). Cells were submitted to the Proteomics and Metabolomics Facility at Colorado State University for fluorescence-activated cell sorting using a MoFlo cell sorter (Beckman Coulter, Brea, CA, USA). For each cat, cells were sorted into four populations: CD4+ (primarily CD4+ T cells), B220+ (B cells), CD8+ (primarily CD8+ T cells), and CD14+ (monocytes). Following sorting, cells were analyzed by flow cytometry to determine purity. All populations contained less than 2% contaminating cells, and most contained less than 1%. Cells from each population in each cat were subjected to RNA extraction and quantification of APOBEC3 RNA by qPCR, as described in Section 2.5. Quantification of FIV gag RNA load by qPCR was conducted as described by Zheng et al. [56]. A portion of each cell population was reserved for DNA extraction followed by FIV proviral load quantification by qPCR, as described by Zheng et al. [56].

### 2.9. In Vivo FIV and PLV Co-Infection

Details of the FIV/PLV in vivo co-infection are provided in Sprague et al. [57]. Briefly, twenty-four cats were divided and housed in the following four groups (*n* = 6 per group): (1) cats inoculated with media (Sham), (2) cats inoculated with PLV-1695 only (PLV), (3) cats inoculated with FIV-C36 only (FIV), and (4) cats inoculated with PLV-1695 followed by FIV-C36 one month later (PLV/FIV). Cats in the PLV and PLV/FIV groups received 10^4.7^ (approximately 50,000) TCID_50_ PLV-1695 by intravenous injection, while cats in the Sham and FIV groups received uninfected Mya-1 cell supernatant. Four weeks later, cats in the FIV and PLV/FIV groups received 10^5.3^ (approximately 200,000) TCID_50_ FIV-C36 by intravenous injection, while cats in the Sham and PLV groups received uninfected Mya-1 cell supernatant. Blood samples were obtained by venipuncture of the cephalic vein periodically throughout the study. At study termination, cats were humanely euthanized and tissue samples were collected and frozen at −80 °C.

### 2.10. Statistical Analyses

All statistical analyses were conducted using Prism v. 5.0 (GraphPad Software, La Jolla, CA, USA). For comparison of APOBEC3 (A3) gene expression over time in FIV-infected versus uninfected cells or between groups of infected/uninfected cats, we performed two-way repeated-measures analysis of variance (ANOVA) and examined the time by virus interaction. Bonferroni post-tests were conducted to identify differences at specific time points. Comparison of multiple groups at a single time point was performed using one-way ANOVA, followed by Tukey’s multiple means comparison test. Group differences were considered statistically significant for *p* < 0.05.

## 3. Results

### 3.1. Expression of APOBEC3 Genes in Cat PBMCs and Cell Lines

**Figure 1:**
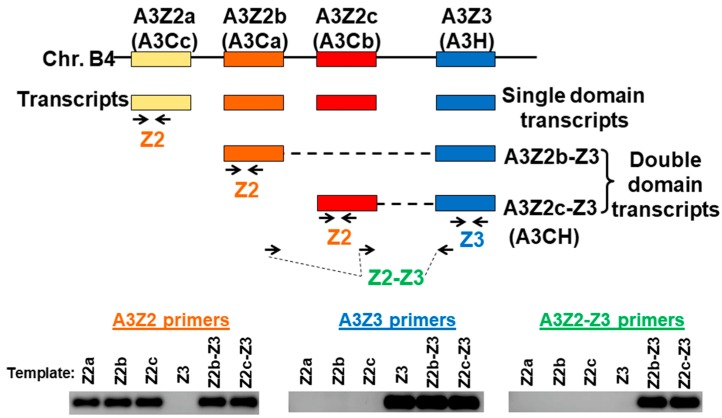
Feline APOBEC3 gene locus and qPCR strategy. Four A3 genes located on cat chromosome B4 include three A3Z2 genes (also referred to as A3C) and one A3Z3 gene (also referred to as A3H). Each of these genes encodes a “single domain” transcript. In addition, double-domain transcripts A3Z2b-Z3 and A3Z2c-Z3 with the Z2 region connected to the Z3 region are produced through alternative splicing [35,39]. Real-time qPCR primers were designed for detection of A3Z2, A3Z3, and A3Z2-Z3 transcripts. To verify primer specificity, a conventional PCR was conducted using the three primers sets to amplify plasmid templates containing A3 transcript sequences. The PCR products from all possible primer–template combinations are shown and demonstrate that all primers were highly specific for the intended target. Since the A3Z2 and A3Z3 primers also detect A3Z2-Z3 expression, we subtracted the A3Z2-Z3 copy number from the A3Z2 and A3Z3 copy numbers for transcript quantitation (see Methods Section 2.5.).

**Figure 2:**
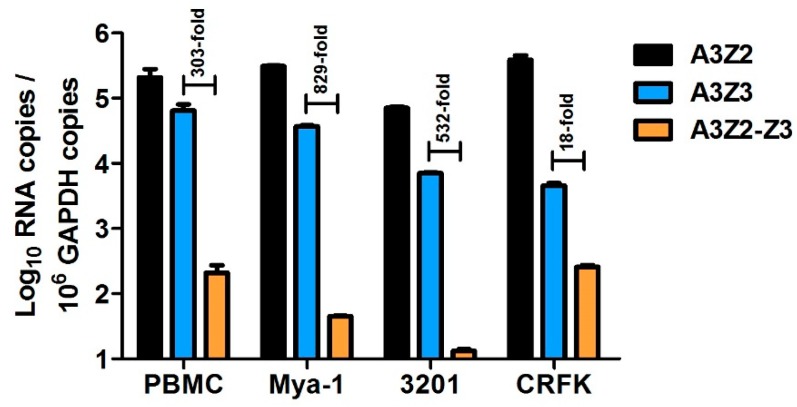
A3 gene expression patterns vary in primary cat peripheral blood mononuclear cells (PBMCs) and cell lines. Within each cell type, differences between A3Z2, A3Z3, and A3Z2-Z3 gene expression were all statistically significant (*p* < 0.001). Experiments were conducted in quadruplicate and error bars represent standard errors of the means. We previously published these data for PBMC, Mya-1, and CRFK cells [64]. Data for 3201 cells is newly published here.

To characterize feline APOBEC3 (A3) gene expression, we utilized three qPCR assays that specifically quantify all known cat A3 cDNAs: (1) all A3Z2 variants, (2) A3Z3, and (3) both A3Z2-Z3 variants (Figure 1). We previously compared expression of A3 genes in cat PBMCs, the Mya-1 feline T cell line, and CRFK feline kidney fibroblast cell line [64]. Here, we additionally determined A3 expression in the 3201 feline T cell line. We found that in all cell types, A3Z2 had the highest total expression followed by A3Z3 (Figure 2). A3Z3 transcripts were expressed at substantially higher levels than A3Z2-Z3 (18 to 829-fold) in all cell types (Figure 2).

### 3.2. Cytokines Regulate APOBEC3 Gene Expression in Vitro

**Figure 3:**
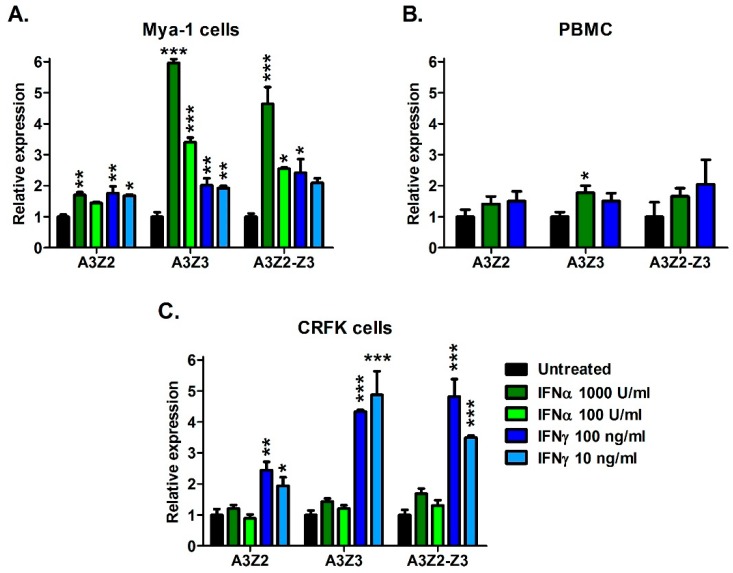
Interferons upregulate A3 mRNAs in a dose-dependent fashion in cat cell lines and PBMCs. (**A**) Mya-1 cells, (**B**) PBMCs, and (**C**) CRFK cells were treated with the indicated concentrations of feline interferon-alpha or feline interferon-gamma and A3 expression was determined 24 h after treatment. A3 expression is plotted and assessed statistically relative to untreated cells (***, *p* < 0.001; **, *p* < 0.01; *, *p* < 0.05). Experiments were conducted in triplicate and error bars represent standard errors of the means.

We next investigated whether A3 gene expression can be regulated by the addition of cellular signaling molecules including cytokines interferon-alpha (IFNα) and interferon-gamma (IFNγ); and mitogens concanavalin A (ConA) and phytohemagglutinin (PHA). Treatment with ConA and PHA for 24 h did not significantly alter A3 gene expression (data not shown). In contrast, IFNα and IFNγ treatment resulted in up to 6-fold upregulation of A3 genes in T cell line Mya-1 and CRFK cells, and to a lesser extent, primary PBMCs (Figure 3). In Mya-1 T cells, treatment with IFNα and IFNγ resulted in upregulation of all A3 genes (Figure 3A). In cat PBMCs, IFNα treatment resulted in upregulation of A3Z3 (Figure 3B). In CRFK cells, treatment with IFNγ resulted in A3 upregulation, while IFNα had no effect (Figure 3C). These data indicate that feline A3 genes can be upregulated by cellular signaling molecules, but the effect varies by molecule and cell type.

### 3.3. APOBEC3 Gene Expression in Cat PBMCs Is Not Altered by in Vitro FIV Infection

**Figure 4:**
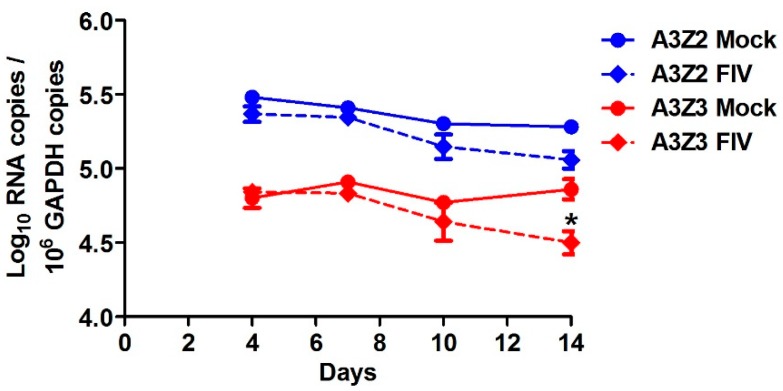
A3 expression in feline immunodeficiency virus (FIV)-infected cat PBMCs in vitro is relatively invariant from uninfected cells. PBMCs from three cats were infected with FIV at MOI 0.001 or mock infected. A3 expression was determined at days 4, 7, 10, and 14 post-infection. The only significant difference between infected and uninfected PBMCs was a reduced level of A3Z3 expression in FIV-infected cells at day 14 (*, *p* < 0.05). There was no difference in A3Z2-Z3 expression between infected and uninfected cells. Error bars represent standard errors of the means.

We examined whether A3 expression in cat PBMCs would be altered by in vitro infection with a virulent host-adapted lentivirus. We challenged PBMCs from specific-pathogen-free (SPF) donor cats with FIV strain C36 [54] and monitored A3 expression in infected versus uninfected cells for two weeks. At 4, 7, and 10 days post-infection there was no difference between FIV-infected and uninfected cells in A3 expression (Figure 4). By 14 days post-infection, viral cytopathic effects were observed in the FIV-infected cell cultures and A3Z3 expression was decreased compared to uninfected cultures (Figure 4).

### 3.4. Lentivirus Infection Does Not Alter in Vivo APOBEC3 Gene Expression in Cat Blood Cells

**Figure 5:**
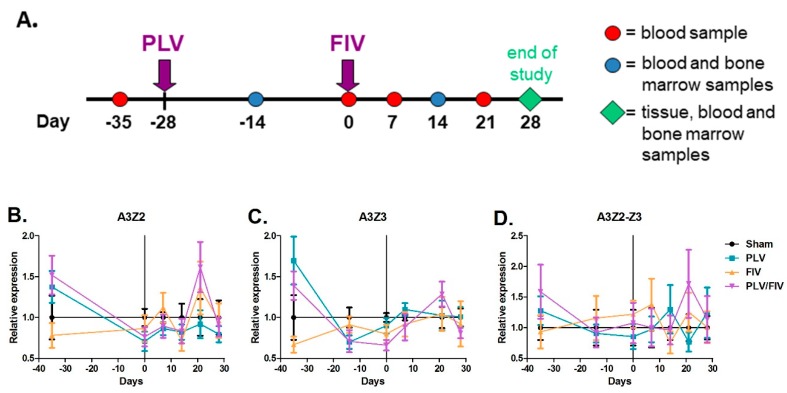
PBMC A3 mRNA expression is not significantly altered following FIV and PLV infection. (**A**) Cats were infected with PLV and four weeks later with FIV (PLV/FIV group, *n* = 6), with PLV only (PLV group, *n* = 6), with FIV only (FIV group, *n* = 6), and sham-infected (Sham, *n* = 6). PBMCs were isolated from all cats prior to infection (day -35), on the day of FIV infection (day 0), and at weekly intervals until four weeks post FIV infection. A3 expression in PBMCs was determined by qPCR and group means with standard error were plotted relative to the sham-infected group for A3Z2 (**B**), A3Z3 (**C**), and A3Z2-Z3 (**D**). There was no significant difference in A3 expression in bulk PBMC mRNA between the Sham group and any of the lentivirus-infected groups at any time point.

We previously found that infection of cats with a non-pathogenic lentivirus from pumas (puma lentivirus, PLV, also known as FIVpco [51]) provided protection from CD4+ T cell depletion during infection with a virulent host-adapted strain of FIV [54]. To further examine immunologic and cellular factors, including A3 expression, that may mediate this novel host protection, we infected cats (*n* = 6) with PLV, followed four weeks later by challenge with FIV (Figure 5A) [57]. Control groups (*n* = 6) included uninfected (sham), PLV infection only, and FIV infection only cats. To determine whether infection with either lentivirus impacted A3 expression in blood cells, we measured expression of A3Z2 (Figure 5B), A3Z3 (Figure 5C), and A3Z2-Z3 (Figure 5D) in peripheral blood mononuclear cells (PBMCs) of all cats prior to and throughout infection/co-infection. There was no significant difference in A3 expression between the sham-infected group and any of the lentivirus-infected groups throughout the infection period (Figure 5), indicating that lentivirus infection does not measurably alter A3 expression in total PBMCs.

### 3.5. APOBEC3 Gene Expression Is Higher in Lymphoid Tissues Than Non-Lymphoid Tissues

**Figure 6:**
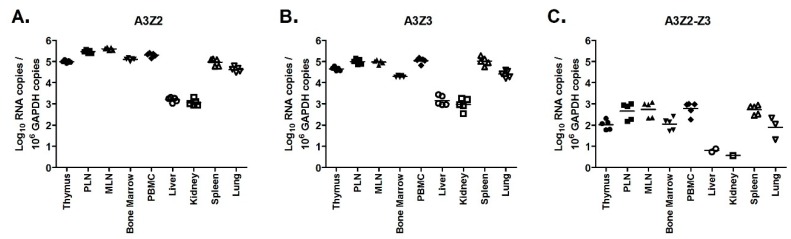
Basal expression of A3 mRNAs is highest in lymphoid tissue. Gene expression in tissues from uninfected cats (*n* = 5) was determined for A3Z2 (**A**), A3Z3 (**B**), and A3Z2-Z3 (**C**). PLN, prescapular lymph node; MLN, mesenteric lymph node. Lines indicate mean copy number for each group. Liver and kidney had significantly lower expression than all other tissues for all A3 mRNAs (*p* < 0.05).

To assess normal A3 expression levels in different feline tissues, we tested A3Z2, A3Z3, A3Z2-Z3 levels in a variety of tissues from uninfected (sham) cats. We found that tissues with high lymphoid content (thymus, lymph nodes, bone marrow, PBMC, spleen, and lung) had 10 to >100-fold higher expression of all A3 genes than tissues with low lymphoid content (liver and kidney) (Figure 6). In all tissues, A3Z2 and A3Z3 had greater than 100-fold higher expression than A3Z2-Z3 (Figure 6). This finding confirms that A3Z2-Z3 is consistently expressed at low levels in cats and, therefore, may not play a major role in cellular host defense. Further, as anticipated, lymphoid cells are the primary cells with relatively high basal A3 expression.

### 3.6. FIV Infection Upregulates APOBEC3 Gene Expression in Thymus, but Not Other Lymphoid Tissues

**Figure 7:**
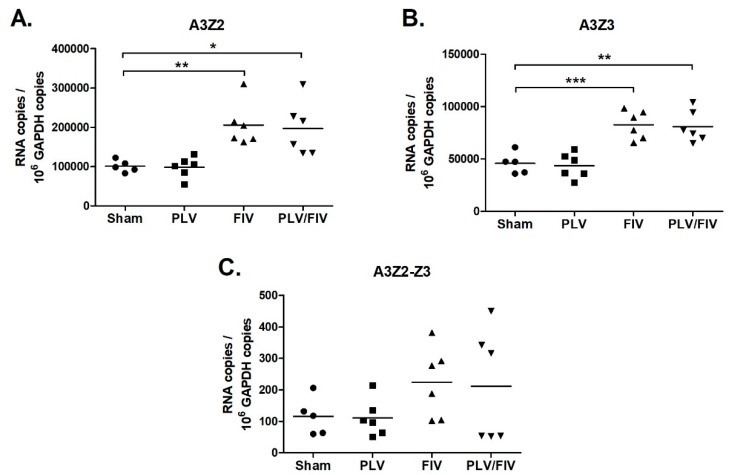
FIV-infected cats have increased A3Z2 and A3Z3 expression in thymus. Gene expression in cat thymus tissue was determined for A3Z2 (**A**), A3Z3 (**B**), and A3Z2-Z3 (**C**). Thymus from cats infected with FIV (FIV and FIV/PLV groups) had increased A3Z2 and A3Z3 mRNA compared to Sham-infected cats (***, *p* < 0.001; **, *p* < 0.01; *, *p* < 0.05). The trend toward greater A3Z2-Z3 expression in FIV-infected cats was not statistically significant. Lines indicate mean copy number for each group.

While lentiviral infection did not alter A3 expression in blood cells (Figure 5), we considered whether infection might alter A3 expression in other lymphoid tissues that are major targets of lentiviral replication. To investigate this possibility, we measured A3 expression in thymus, bone marrow, prescapular lymph nodes, and mesenteric lymph nodes of uninfected, PLV-infected, FIV-infected, and PLV/FIV co-infected cats. In bone marrow and lymph nodes, A3 expression in lentivirus-infected cats was similar to that of uninfected cats (Figure S1). In thymus, A3Z2 and A3Z3 expression in both FIV and PLV/FIV-infected cats was increased compared to uninfected controls (Figure 7). The thymus was also the site of highest FIV viral load in these animals [57]. There was a trend toward increased A3Z2-Z3 expression in FIV and PLV/FIV-infected cats, but it was not statistically significant (Figure 7). Cats infected with PLV alone had similar A3 expression to uninfected controls (Figure 7), indicating that infection with FIV and not PLV caused increased thymic A3 expression.

### 3.7. Effect of FIV Infection on APOBEC3 Expression in Major Blood Cell Sub-Populations

**Figure 8:**
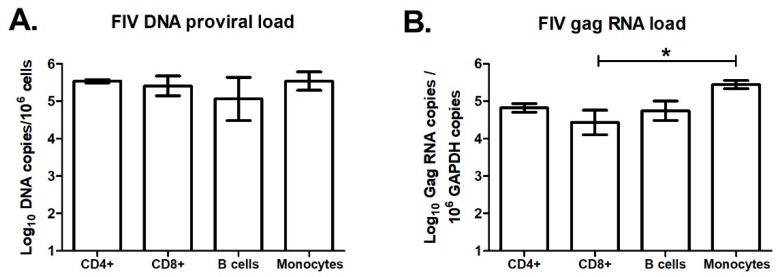
FIV is pancytotropic and replicates in monocytes and lymphocytes. PBMCs were isolated from three chronically FIV-infected cats (18-weeks post infection) and cells were sorted by fluorescence-activated cell sorting (FACS) into CD4+, CD8+, B cell (B220+), and monocyte (CD14+) subsets. FIV DNA proviral load (**A**) and FIV gag RNA load (**B**) in each of these cell subsets were determined by qPCR. CD8+ cells had significantly lower FIV gag RNA than monocytes (*, *p* < 0.05). All other differences between groups were non-significant. Error bars represent standard errors of the means.

**Figure 9:**
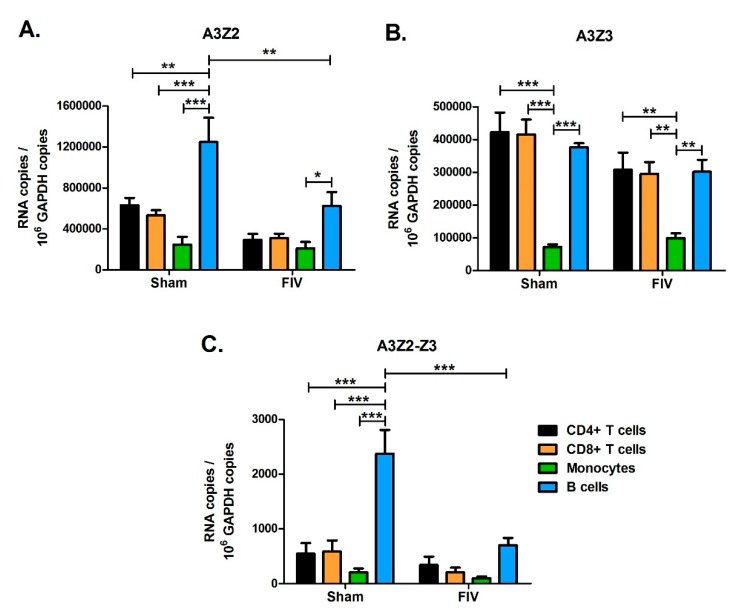
A3 expression patterns in leukocyte subtypes from FIV and Sham-infected cats. PBMCs were isolated from three chronically FIV-infected cats (18-weeks post infection) and cells were sorted by FACS into CD4+, CD8+, B cell (B220+), and monocyte (CD14+) subsets. Expression of A3Z2 (**A**), A3Z3 (**B**), and A3Z2-Z3 (**C**) mRNA was determined for each cell subset. Expression levels were compared between cell types, e.g., B cells versus monocytes, and between Sham- and FIV-infected animals within the same cell type, e.g., Sham B cells versus FIV B cells. Statistically significant group mean differences are displayed for all significant comparisons (***, *p* < 0.001; **, *p* < 0.01; *, *p* < 0.05). Comparison of cell type indicated that B cells had higher A3Z2 and A3Z2-Z3 expression than T cells and monocytes, while monocytes had lower A3Z3 expression than lymphocytes. Comparison of Sham- and FIV-infected cats indicated that FIV-infected cats had lower A3Z2 and A3Z2-Z3 expression in B cells; all other differences between Sham and FIV were not statistically significant. Error bars represent standard errors of the means.

While A3 expression levels in total PBMCs of lentivirus infected cats were similar to levels in uninfected cats (Figure 5), it is possible that A3 genes might be differentially regulated in different blood cell subpopulations. To investigate this possibility, we designed an experiment to determine A3 expression levels in major blood cell subpopulations from uninfected and FIV-infected cats. We infected three cats with FIV-C36 and sham-infected three additional cats by intravenous injection. At 18-weeks post-infection, during the chronic infection phase, we used fluorescence-activated cell sorting (FACS) to isolate CD4+ T cells, CD8+ T cells, B cells, and monocytes from blood of uninfected cats and infected cats. We determined FIV-C36 DNA proviral loads and gag RNA levels for each cell type by qPCR. FIV DNA and RNA levels were similar among the four cell types, with the exception that monocytes had significantly higher gag RNA than CD8+ T cells (Figure 8). We then determined A3 mRNA expression in each cell type and found that the different A3 genes were preferentially expressed in different blood cell subsets. A3Z2 and A3Z2-Z3 expression in uninfected cats was higher for B cells compared to T cells and monocytes (Figure 9). In contrast, A3Z3 was expressed at similar levels in B cells and T cells, but at much lower levels in monocytes (Figure 9). There was no significant effect of FIV infection on A3 expression in T cells or monocytes (Figure 9). For B cells, FIV infection was associated with reduced expression of A3Z2 and A3Z2-Z3, but no change in A3Z3 expression (Figure 9). Thus, FIV infection clearly does not increase A3 mRNA expression in the major blood cell subpopulations.

## 4. Discussion

Previous studies have established evidence for the innate immune system as a primary modulator of lentiviral infection in cats, with cellular A3 enzymes hypothesized to play an essential role [56,57]. Importantly, the innate immune response has been implicated in partial protection against virulent lentiviral infection conferred by primary exposure to an apathogenic lentivirus [54,55]. Goals of the current study were 3-fold. First, we aimed to establish baseline expression levels of feline A3 genes and compare across tissue and cell types. Second, we sought to examine the effect of regulatory molecules (cytokines, mitogens, and lentiviruses) on A3 expression in cultured cells. Lastly, we compared the effects of host-adapted FIV and non-adapted PLV on A3 expression across tissue and cell types in vivo and in different leukocyte populations.

We measured in vivo expression of all feline A3 genes in PBMCs prior to and throughout infection and found that A3 expression is not dramatically altered by infection with host-adapted FIV or non-adapted PLV. Global measurement of A3 expression in PMBCs revealed no significant difference between infected and non-infected cats. We additionally investigated the possibility that global measurement of PBMC expression could mask differential regulation across specific blood cell subpopulations. Although we found that basal A3 gene expression in blood is cell-type specific in cats, FIV infection consistently had either no measurable effect or corresponded to reduced A3 expression. Thus, we did not find evidence for upregulation of A3 expression in major blood cell subpopulations as a result of FIV infection. Given this finding, it is unlikely that A3 expression is the primary mechanism that mediates PLV-induced resistance to FIV.

In addition to measuring A3 expression in the peripheral blood, we measured A3 expression in tissue sites of active viral replication and with varying immigrant populations of infected cells. We found that FIV-infected thymus had increased A3 expression. Given the tropism of FIV for T cells, thymus is a major target organ serving as both a putative site of immune activation and a tissue reservoir for FIV-infected cells. We previously noted increased IFN-γ expression in the thymus in FIV/PLV co-infected cats as compared to single FIV-infected cats [56]. Similarly, we have shown that IFN-γ expression in PBMCs is significantly higher in co-infected cats as compared to those with single FIV infections [54,57]. These findings evoke a regulatory role for IFN-γ and we, therefore, hypothesized that interferon mediates enhanced expression of feline A3 in response to FIV infection. We found that both IFN-γ and IFN-α were able to upregulate feline A3 expression in vitro to varying degrees in different cell types. This finding establishes that feline A3 expression can, at least in some circumstances, be regulated by exogenous signals and supports the idea that IFN production in response to FIV infection could upregulate A3. In FIV-infected cats, we did not find a significant change in A3 expression levels in PBMCs and most tissues. The exception was thymus, the site of highest FIV viral load in these cats [57], which had increased A3 expression in FIV-infected cats. Since FIV infection does not appear to directly upregulate A3 in vitro, it seems likely that A3 upregulation in the thymus may be due to FIV-infection-induced signaling molecules such as IFN-γ or IFN-α. While A3 is not globally upregulated in cats in response to FIV-induced cytokines, IFN expression is likely tightly controlled locally and the methods used here may not have been sensitive enough to detect low-level upregulated A3 expression in small populations of cells.

As expected from previous studies in humans [18,22], lymphoid tissues of cats had higher expression of A3 than non-lymphoid tissues. Somewhat surprisingly, A3Z2-Z3 transcripts were present in very low abundance in all tissues and cells. This finding suggests that A3Z2-Z3 may be of limited importance to the antiviral restriction system and supports prior evidence for A3Z3 as the primary A3 variant providing defense against lentiviruses, despite the fact that in vitro studies have demonstrated that anti-FIV activity is conferred by A3Z2-Z3 [35]. This finding may also be relevant to FeLV as A3Z2-Z3 has been shown to significantly reduce FeLV in vitro infectivity while A3Z3 had only marginal impact on FeLV in vitro infectivity [35]. It is possible A3Z2-Z3 transcripts could be produced predominantly by a specific cell type not identified in the current study or A3Z2-Z3 protein quantity may be higher than what would be expected based on the very low level of mRNA as suggested by Zhang et al. [48]. Future studies comparing A3 protein levels in cells and tissues will be useful to verify expression based on mRNA levels.

In this study, we found that A3 expression was generally not enhanced in cats in response to infection with domestic-cat- or puma-adapted FIV (puma lentivirus, PLV). Since previous studies indicate that A3 proteins likely play a role in limiting and controlling PLV infection of domestic cats [53,66], these findings suggest that constitutive, rather than regulated levels of cat A3 can be sufficient to inhibit lentiviral cross-species infection. Lentiviral Vif may evolve to counter A3 restriction, resulting in competent infection and enhanced virulence, so the ultimate outcome of an evolutionary conflict between cat A3 and a non-adapted FIV is dependent upon both host and viral adaptation [16,45,67]. Even in the case of a host-adapted lentivirus, there is likely a dynamic balance between A3-mediated restriction and Vif-mediated protection. For example, our work with chimeric FIVs demonstrated that the high replication rate of a virulent FIV strain (C36) relative to a less virulent strain (PPR) was conferred largely by Vif [64]. Viruses that differed only in Vif had different rates of replication in cells expressing A3 genes but knockdown of A3Z3 and A3Z2-Z3 in cells resulted in equalization of replication between viruses. Thus, the balance between A3-mediated restriction and Vif-mediated protection is likely controlled less by the capacity of the virus to alter A3 expression and more by the capacity of Vif to counteract A3.

We found that basal levels of A3 expression varied between subtypes of lymphocytes that are all capable of supporting FIV replication. Most notably, monocytes expressed substantially lower amounts of all A3 subtypes (Figure 9), although all cells were capable of supporting active FIV replication (Figure 8), suggesting limited impact of A3 expression on cell tropism for a host-adapted virus. It is feasible that A3 variant expression levels may dictate cell tropism of non-adapted FIV (i.e., PLV) replication and implies that viral evolution rates may vary by cell type. Collectively, our findings suggest that lentiviral replication competence in cats is influenced primarily by the capacity of Vif to counteract A3 and less so by modulation of A3 expression. The findings of this study also demonstrate the variable response of A3 gene expression to cellular signaling molecules, the variable expression of A3 in different blood cell types, and highlight the complex interplay between specific effector molecules and cells of innate immunophenotype.

## Supplementary Materials

The following are available online at https://www.mdpi.com/1999-4915/11/9/831/s1, Figure S1: A3 mRNA expression in important tissue targets of lentivirus infection is not altered by FIV or PLV infection.
