# Supplementary material for: Expression of APOBEC3 Lentiviral Restriction Factors in Cats
Source: Viruses. 2019 Sep 7;11(9):831. doi: 10.3390/v11090831 (PMC6783916; doi:10.3390/v11090831)
Supplement: Supplementary file 1 [file viruses-11-00831-s001.pdf]

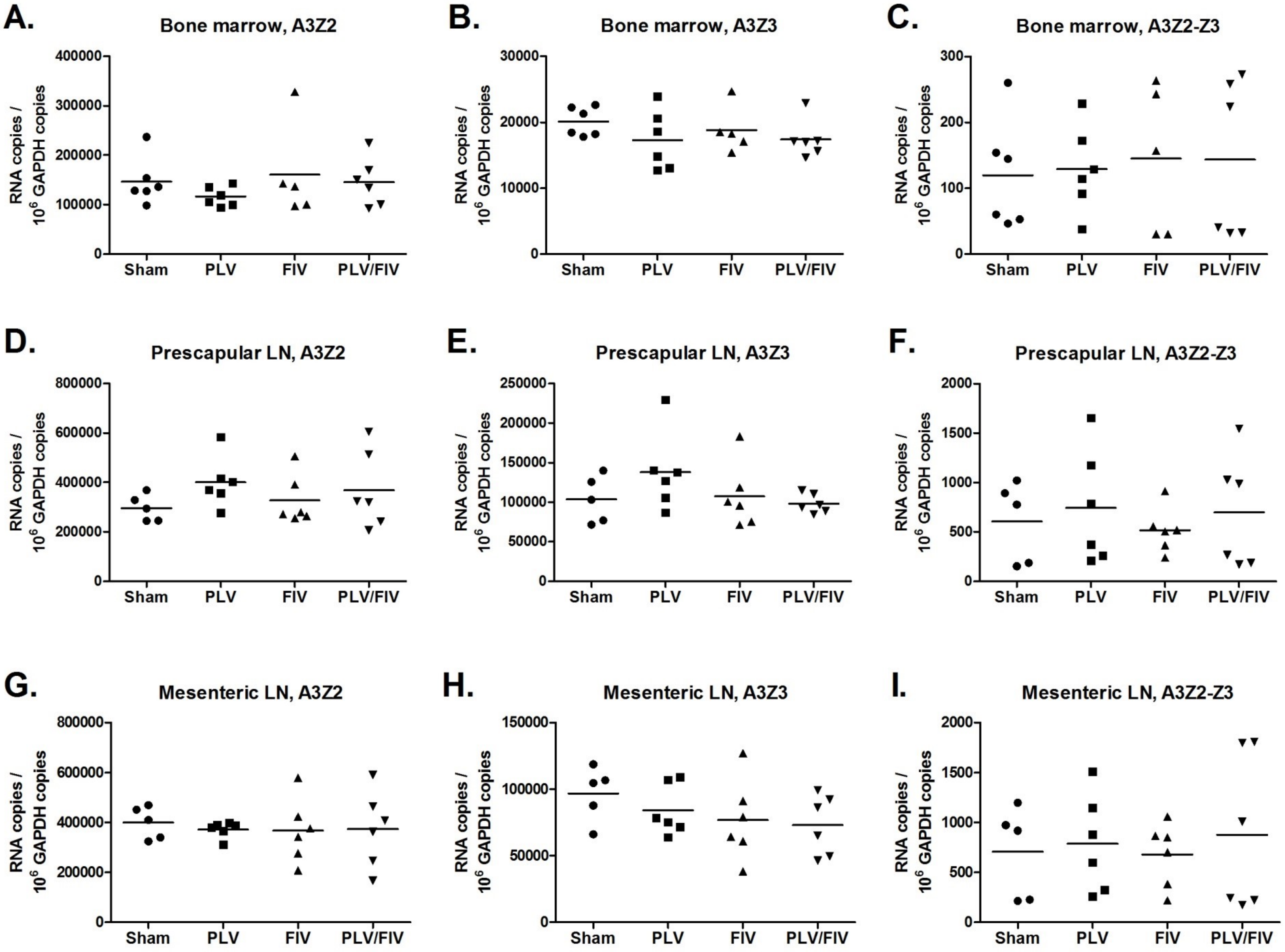

Supplementary Figure 1. A3 mRNA expression in important tissue targets of lentivirus infection is not altered by FIV or PLV infection. RNA copy number of A3Z2, A3Z3, and A3Z2-Z3 was determined for bone marrow (A, B, C), prescapular lymph node (D, E, F), and mesenteric lymph node (G, H, I), respectively. Lines indicate mean copy number for each group.
